# Sensors for rate responsive pacing

**Published:** 2004-07-01

**Authors:** Simonetta Dell'Orto, Paolo Valli, Enrico Maria Greco

**Affiliations:** Unità Operativa Cardiologia, Ospedale Uboldo, Azienda Ospedaliera di Melegnano; Cernusco sul Naviglio, Milano, Italy

**Keywords:** rate responsive pacing, activity sensors, metabolic sensors, physical activity, biventricular pacing, implantable cardioverter defibrillator

## Abstract

Advances in pacemaker technology in the 1980s have generated a wide variety of complex multiprogrammable pacemakers and pacing modes. The aim of the present review is to address the different rate responsive pacing modalities presently available in respect to physiological situations and pathological conditions. Rate adaptive pacing has been shown to improve exercise capacity in patients with chronotropic incompetence. A number of activity and metabolic sensors have been proposed and used for rate control. However, all sensors used to optimize pacing rate metabolic demands show typical limitations. To overcome these weaknesses the use of two sensors has been proposed. Indeed an unspecific but fast reacting sensor is combined with a more specific but slower metabolic one. Clinical studies have demonstrated that this methodology is suitable to reproduce normal sinus behavior during different types and loads of exercise. Sensor combinations require adequate sensor blending and cross checking possibly controlled by automatic algorithms for sensors optimization and simplicity of programming. Assessment and possibly deactivation of some automatic functions should be also possible to maximize benefits from the dual sensor system in particular conditions. This is of special relevance in patient whose myocardial contractility is limited such as in subjects with implantable defibrillators and biventricular pacemakers. The concept of closed loop pacing, implementing a negative feedback relating pacing rate and the control signal, will provide new opportunities to optimize dual-sensors system and deserves further investigation. The integration of rate adaptive pacing into defibrillators is the natural consequence of technical evolution.

The advances in pacemaker technology in the 1980s have generated a wide variety of complex multiprogrammable pacemakers and pacing modes. Pacing systems can be classified as (1) single lead ventricular, such as VVI or ventricular demand; (2) atrial based, single lead, such as AAI or atrial demand; or dual chamber, such as DDD, which senses and paces from atrial and ventricular chambers. These pacing modalities may or may not be rate adaptive or sensor-driven. In the pacemaker code, rate adaptive pacemakers are designated with a fourth letter R, for example a rate adaptive DDD becomes DDDR. Rate adaptive pacemakers are useful for patients who cannot increase their heart rate appropriately on exercise.

The goal of new technologies is to come as close as possible to sinus node electrical activity, combining the need of regular ventricular rate to a better quality of life. In this context the clinical benefit of increasing ventricular rate, and consequently cardiac output, during physical activity in patients with “chronotropic incompetence” consequent to Sick Sinus Syndrome or advanced AV block, has been established by different studies [1-7].

The ACC/AHA guidelines [8] define chronotropic incompetence as failure to achieve a heart rate of 100 beats/min at maximal exertion. From the practical standpoint heart rate at a given metabolic load should mirror the line that describes the correlation of heart rate to metabolic demand for age, sex, and weight matched control group [8]. This definition avoids a fixed cutoff heart rate as a criterion, and considers the trend of heart rate during different intensity of exercise as the frame of reference [9-11].

## Fixed rate versus rate responsive pacing

Many studies have shown that during exercise, an increase in the pacing rate provided by VVIR, VDD, DDD or DDDR modes augments the cardiac output, achieved workload, and duration of exercise more than does fixed-frequency VVI pacing in patients with both normal or impaired LV function [2-7,12-15]. Beside superior hemodynamic effects, there is an increase in maximum oxygen consumption, a reduction in arteriovenous oxygen difference and an increase in subject well-being. Moreover, the acute hemodynamic advantage is retained on a long term basis: by 6 and 12 months after implantation, dual chamber and rate adaptive pacemakers may further augment LV function, reduce heart size and improve ventricular performance compared with results in the immediate postoperative period [16-17].

When patients with complete AV block exercise, the sinus rate is significantly higher with VVI pacing than during dual chamber or VVIR modes, a response possibly reflecting the increased activity of the sympathetic nervous system when pacing is set on VVI mode [18-19]. Indeed, coronary sinus norepinephrine is higher in patients with VVI pacemaker during exercise. The increase in catecholamine on exercise during VVI pacing is likely to be related to the need of improving contractility and therefore cardiac output to compensate the lack of rate response. This enhanced cardiac sympathetic activity may eventually produce an adverse effect on LV function, with the possible development of congestive heart failure and arrhythmias.

In patients with atrial chronotropic incompetence, VVIR and DDDR pacing modes are clearly superior to the DDD mode in terms of exercise performance, because the sensor increases the pacing rate according to metabolic needs. Most studies [20-21]. on patients with atrial chronotropic incompetence and DDDR pacemakers have shown superior hemodynamic performance on exercise, and patients prefer the DDDR mode to the VVIR mode.

Moreover, preliminary data suggest that rate adaptive AAIR and DDDR modes may be more efficacious in preventing atrial arrhythmias than their non rate adaptive counterparts in Sick Sinus Syndrome [22-24]. DDDR pacemakers may prevent arrhythmias by eliminating the relative bradycardia noted during exercise in patients with non-adaptive devices, when excessive catecholamine release may increase the likelihood of atrial arrhythmias.

## Rate responsiveness

Rate-adaptive pacing has been designed to increase heart rate according to metabolic needs during physical, mental or emotional activity.  Rate responsive pacemakers control heart rate by sensing physiological or nonphysiological signals other than atrial rate.

Ideally the rate adaptive sensors should reproduce the sinus node as close as possible; therefore some definite properties must be accomplished: (1) the chronotropic output should respond as promptly as the normal sinus node. (2) Sensors should perform a highly specific and sensitive detection of the need of increasing heart rate. (3) These latter have also to be proportional to metabolic demand. (4) Rate decay during recovery after exercise should match metabolic needs (i. e. fast after short exercise but prolonged after longer and maximal exercise in response to an oxygen debt or in pathological conditions like heart failure); (5) it should ideally operate in a closed loop system, making rate adaptive pacing also insensitive to inputs not heart related. Finally, (6) dedicated sensors should avoid undesiderable overpacing and the need of complex programming.

Different parameters have been investigated for controlling the pacemaker rate: oxygen saturation [25], venous pH [26], QT interval [27,28], body motion [28], respiratory rate [29], stroke volume [30], central venous temperature [31-33], minute ventilation [34], peak endocardial acceleration [35], and changes of the right ventricular impedance during the cardiac cycle (CLS, closed loop stimulation) [36]. Clinical studies have outlined advantages and limitations of the different sensed parameters. Finally complexity of implanting and programming, the evidence of instability related to influence of  external conditions or concomitant disease, have defined the inadequacy of some parameters to the required characteristics, and only some of these indicators are still used as single or dual sensor technology.

## Single sensor technology

Activity sensors are the older and more widely used. The working modality is based on the relationship between activity and heart rate. Activity may be acknowledged either by a piezoelectric crystal, which recognizes the muscular pressure waves, produced by physical activity and convert them to an electrical signal sent to the pacemaker, or by an accelerometer that identifies the postural changes and the body movements related to physical activity.

Activity sensors offer rapid response to exercise by assessing body vibrations or movements. A rapid response plays an important role in “burst activity” during daily life. Fast reaction to termination of short exercise and technical simplicity that allows for instance to tailor the rate response (RR) to the single patient with proper treadmill protocols, [37] represent further advantages of this sensor type. However, after longer exercise, an oxygen debt may require a sustained rate increase, which is not provided by activity sensors during recovery because these sensors are unable to recognize the oxygen debt. Moreover, low specificity with inappropriate rate increase in conditions like laughing, coughing, driving, the fact that activity sensors does not respond to activity not related to body movements (isometric exercise, mental stress, or metabolic inadequacy consequent to pathologic conditions), and the possible mismatch between exercise intensity and rate increase, represent the main limitations of activity sensors.

Metabolic sensors, based on QT interval, minute ventilation (MV) or peak endocardial acceleration, provide pacing rates more closely and specifically related to physical and mental stress requirements.

Minute ventilation, the product of respiratory rate and tidal volume, is a physiological indicator that has been shown to be correlated with metabolic demand [38,39]. This parameter, which also correlate linearly with heart rate [40,41], can be derived from variations in transthoracic impedance signal. RR pacemakers, using impedance MV sensors, change the pacing rate in response to the variations in the patients MV.

Limitations of the MV sensor include the lower reliability in patients with obstructive pulmonary disease, false positive reaction in hyperventilation or interference with cardiac monitors [42] and posture [43]. Sensors using QT interval variations [27,44] are based on the finding that physical activity and circulating catecholamine produce shortening of the QT interval. These sensors are highly specific; furnish sustained increase of sensor-driven heart rate during post-exercise recovery to compensate for an oxygen debt, and are responsive to mental stress. However, measurement of evoked QT interval may be unreliable in T wave undersensing; it can not be used in patients with acute myocardial infarction, is affected by drugs, electrolyte disturbances and increased circulating catecholamine, a common condition in patients with congestive heart failure. Because it requires ventricular pacing, it can not be used in AAIR mode.

More recently, a sensor that assesses mechanical vibrations generated by the myocardium during the isovolumetric contraction phase (peak endocardial acceleration [PEA]), has been developed. A micro accelerometer is housed inside a rigid, perfectly hermetic capsule inserted in the tip of a standard unipolar pacing lead. The rigidity of the capsule prevents the generation of artifacts that may arise from compression of the electrode by the cardiac muscle during contraction. Therefore, the sensor is only sensitive to the inertial forces generated by myocardium movements. An associated electronic circuit pre-processes the signal to ensure its correct transmission trough the catheter.

Experimental and clinical trials have shown that ∆PEA is correlated with dP/dT max [35,45,46] and is consequently related to contractile function.  ∆PEA dynamic monitoring has been shown to provide fast pacing rate responses with long term performance of sensor lead and effective and rapid RR tailoring [35] [47], also in patients with heart failure and wide QRS [48]. Moreover Peak Endocardial Acceleration assessment allows AV delay automatic optimization in DDDR pacing that can be calculated directly from the device using a time saving procedure [49]. Experimental data in animals indicate that PEA monitoring is feasible also during atrial fibrillation [50].

PEA sensor is usually combined with activity sensors, however, in view of its rapid and appropriate heart rate response in different conditions, the possibility to use PEA sensor as a single sensor should also be considered.

## Dual sensors pacemakers

As previously described no single sensor can reproduce sinus node behavior in all the different activities of daily life. To overcome these limitations a possible option is to combine two different sensors. Rate modulation has to be considered at three different levels: (1) short term response for effort or emotions; (2) medium term adaptation for circadian variation of heart rate during day- and night-time; and (3) long term regulation to obtain different rate variations according to rest and activity periods [51].

Combining different sensors might more closely mimic intrinsic heart rate, if the chosen sensors are complementary. The most common combination include association of an activity sensor giving a rapid response for light or for short duration exercise, and a metabolic sensor, e.g. QT interval or minute ventilation (MV) that provides a delayed but proportional and stable acceleration to sustained exercise and deceleration during recovery [51]. Another option in rate response devices is to obtain circadian heart rate variation with two different hourly mean rates during day and night. Physiologic sensors and activity sensors could provide rate variations based on signal sensor solicitation. Two lower heart rates are programmed for daytime and nighttime. When the sensor is constantly solicited, the daytime lower rate is used. On the contrary, when the signal sensor level is low for a consistent period of time, the device switches on nighttime lower rate. Metabolic sensors could provide a modulation of the algorithm curve slope according to the long term activity.

## Sensors optimization

Independently of the choice of sensors and mode of integration, algorithms for sensors optimization determine the performance of dual sensor rate adaptive pacemakers.

 Combining sensors with different rate responses requires adequate blending of respective sensor activities. Blending can be performed at signal production. The resulting signal transmitted to the algorithm is a mixture of a percentage of activity sensor signals (0%…10%) with an inverse percentage of non activity sensor signals (100%…0%). This blending modality is used for instance in the Vitatron device, combining QT interval and activity in 5 different possibility. This blended signal is transmitted to the algorithm working with a variable automatic slope [51].

Another possibility for sensor blending is priorization as in Medtronik Kappa 400: the activity sensor initially accelerates from the lower rate to a plateau (programmable 90-95 b/min). The rate returns to the lower rate if activity stops, or is proportionally increased from the plateau up to the sensor maximal programmed rate, if the minute ventilation sensor activates. MV sensor is then in charge during the recovery rate decrease.

Sensors cross checking are used to avoid inappropriate rate increase. During crosscheck both sensors can control each other and the pacing rate will only be changed if both or a predominant sensor agrees. For example, after administration of a drug that shortens the QT interval, a QT interval sensor would indicate the need for rate increase, but the pacing rate would not change because the activity sensor is not activated. Conversely, passively tapping on the device would activate the activity sensor and indicate a rate increase, but the pacing rate would not be modified because the QT-interval sensor would not be activated by this manouver.

## Algorithms for optimization of dual sensor performance

Algorithms for sensors optimization determine the performance of dual-sensor rate-adaptive pacing systems. Automatic setting has been developed in complex pacemakers to simplify programming and optimize time. It is necessary for physicians to be able to verify that rate adaptive pacemakers respond correctly, according to the patient’s need. Nevertheless, even though these systems are generally reliable [47], manual access to sensor programmability is important and should be performed by physicians with a thorough knowledge of the sensors capabilities. In addition, a specific apparatus for O_2_ consumption measurement during physical exercise (cardiopulmonary stress test) should be used. Alternatively an algorithm computing the best correlation between heart rate and metabolic needs, such as  Pacing Rate Profile Software (PRPS), ought to be used [47].

Future devices may provide the opportunity to use physiologic sensors to monitor cardiac function and to adapt pacemaker function to assist therapy for associated disorders. Multisensor devices can be used for cardiac rehabilitation in pacemaker dependent patients, particularly the elderly and affected by cardiopulmonary disease. These patients deserve a physically and psychologically autonomous life style, which may be accomplished by using two rehabilitation methods: (1) the set up of appropriate rate response; and (2) the institution of aerobic training programs [52] for in- and outpatients.

Moreover, the integration of rate adaptive pacing into biventricular pacemakers and implantable defibrillators is a natural consequence of technical evolution. The rationale for the use of rate adaptive pacing in implantable defibrillators is the same as for pacemaker.  In normal heart, an increase in oxygen uptake from 3 to 50 mL/kg per minute in a healthy subject is due to an increase of oxygen extraction rate, stroke volume (by a factor of 1.5) and heart rate (by a factor of 3). This allows a 4-5 fold increase in cardiac output compared to resting values. In the large majority of patients receiving an implantable defibrillator, the contractility reserve is limited. Therefore, an increase in cardiac output is strictly related to the possibility of increasing heart rate. Concomitant therapy with beta blockers, amiodarone or other antiarrhythmic drugs may also impair the chronotropic response. This makes the issue of chronotropic competence crucial in patients with biventricular stimulation and implantable defibrillator despite the fact that only 20% of these latter need primarily a cardiac pacing [8].

The concept of closed loop pacing should be the next step in future technical developments. Sensors that could be used in a closed loop system, indicating whether heart rate is adequate to a given metabolic situation, are endocardial accelerometers and sensors using impedance derived ventricular signals. It might take some time to test technical feasibility and clinical reliability of those closed loop systems before they will be implemented in cardiac pacemakers and implantable defibrillators or biventricular pacemakers.
